# 5-α-reductase inhibitors and the risk of suicide and neuropsychiatric symptoms: a meta-analysis of observational studies

**DOI:** 10.3389/fruro.2026.1881635

**Published:** 2026-10-02

**Authors:** Federica Fraenza, Valerio Liguori, Ciro Pentella, Consiglia Riccardi, Francesca Futura Bernardi, Annalisa Capuano, Cristina Scavone

**Affiliations:** 1 Department of Experimental Medicine, University of Campania “Luigi Vanvitelli”, Naples, Italy; 2 Campania Regional Centre for Pharmacovigilance and Pharmacoepidemiology, University of Campania “Luigi Vanvitelli”, Naples, Italy; 3 Coordination of the Regional Health System, General Directorate for Health Protection, Department of Experimental Medicine, University of Campania Luigi Vanvitelli, Naples, Italy; 4 Department of Life Science, Health, and Health Professions, Link Campus University, Rome, Italy

**Keywords:** 5-ARI, AGA, BPH, meta-analysis, neuropsychiatric events, observational studies, systematic review

## Abstract

**Introduction:**

5-alpha-reductase inhibitors (5-ARIs), such as finasteride and dutasteride, are effective in reducing prostate volume and urinary symptoms; however, these drugs are associated with specific adverse events, including suicidal ideation, anxiety, and self-harm.

**Methods:**

A systematic review and meta-analysis of observational studies was conducted in PubMed, Embase and Web of Science on July 16, 2025, with no time restrictions. Studies involved individuals diagnosed with benign prostatic hyperplasia (BPH) or androgenetic alopecia (AGA), and experiencing suicide and neuropsychiatric events, including anxiety, depression and intentional self-harm (ISH) during the treatment with 5-ARIs. This study followed the PRISMA statement.

**Results:**

Eleven studies were included in the systematic review, of which nine were eligible for meta-analysis. Comparisons included 5-ARI users versus non-users or alpha-blocker users. Overall, no statistically significant differences were observed between groups (HR 1.07, 95% CI: 0.76–1.51), with substantial heterogeneity across studies (I² = 59%).

**Conclusion:**

This meta-analysis found no significant association between 5-ARIs and the risk of suicide or neuropsychiatric events in patients with benign prostatic hyperplasia or androgenetic alopecia. Nevertheless, given regulatory concerns regarding suicidal ideation, continued real-world monitoring through active surveillance and prospective observational studies remain warranted.

**Systematic Review Registration:**

https://www.crd.york.ac.uk/PROSPERO/view/CRD420251106035, identifier CRD420251106035.

## 1 Introduction

5-α-reductase inhibitors (5-ARIs), which include finasteride and dutasteride, work by preventing the conversion of testosterone into the more potent androgen dihydrotestosterone (DHT). Finasteride inhibits type II and, to a lesser extent, type III isoenzymes of 5α-reductase, thereby reducing DHT levels in serum and tissue (1). Similarly, dutasteride inhibits 5α-reductase, targeting both type I and type II isoenzymes, resulting in a more significant reduction of DHT in target tissues and serum compared to finasteride (90-94% vs. 65-70%) (2). Benign prostatic hyperplasia (BPH) is the primary condition for which both drugs are prescribed. Their ability to decrease prostate volume reduces the risk of BPH complications while improving urine flow and alleviating symptoms such as those measured by the International Prostate Symptom Score (3). At lower doses, finasteride is also approved for the treatment of androgenetic alopecia (AGA), or male pattern hair loss, where it enhances hair density and prevents hair loss (4). Both medications decrease prostate size, enhance urine flow metrics, and alleviate BPH symptoms, although dutasteride has demonstrated a larger reduction in symptom scores and prostate-specific antigen (PSA) levels compared to finasteride (5). Lastly, premarketing and postmarketing clinical studies show that when administered to treat androgenetic alopecia, dutasteride improves hair thickness and count more than finasteride (6, 7).

Regarding their safety profiles, both drugs are commonly associated with sexual adverse events (AEs) such as erectile dysfunction, decreased libido and ejaculatory volume. However, in recent years, there has been increased interest in the neuropsychiatric safety profile of 5-ARIs that seem to be associated with suicidal thoughts, anxiety, despair, and self-harm (8), becoming a matter of concern of the Pharmacovigilance Risk Assessment Committee (PRAC) of the European Medicines Agency (EMA). After reviewing the available data on finasteride tablets across the EU, the PRAC verified that suicidal thoughts are a negative effect of 5-ARIs and suggested measures to lower the risk of suicidal ideation associated with finasteride and dutasteride (9, 10) that were approved by the Coordination Group for Mutual Recognition and Decentralised Procedures - Human (CMDh) on June 19, 2025. Considering these recent safety concerns related to 5-ARIs, we carried out a systematic review and meta-analysis of observational studies to identify the effects of these drugs on the risk of suicide (primary objective) and on the risk of neuropsychiatric symptoms, including anxiety, depression and intentional self-harm (secondary objective).

## 2 Methods

The study was designed and reported with adherence to the Preferred Reporting Items for Systematic Reviews and Meta-analysis (PRISMA) guidelines (11). This systematic review and meta-analysis was registered to the International Prospective Registry of Systematic Reviews (PROSPERO) database and assigned the PROSPERO registration number: CRD420251106035.

### 2.1 Search strategy and study selection

Studies were identified through a comprehensive bibliographic search in three electronic databases (Medline via PubMed, Embase and Web of Science) that was conducted on July 16, 2025, with no time restrictions.

Three authors (FF, CR and CP) used the Medical Subject Headings (MeSH) database to retrieve the synonyms of our search strategy, and the terms were combined using “OR” and “AND” Boolean operators. The search strategy utilized was as follows: (Suicid* OR suicidality OR suicidally OR suicidals OR suicide OR suicides OR suicided OR suiciders OR suicidal behaviors OR attempted suicide OR suicidal ideation OR suicide thinking OR suicidal ideations OR suicidal thoughts OR suicidal thought OR depressed OR depression OR depressions OR depressive disorder OR depression disorder OR depressivity OR depressive OR depressively OR depressiveness OR depressives OR anxiety OR suicidal behaviors OR anxieties OR suicidal behaviors OR anxieties OR safety) AND (5 alpha reductase inhibitors OR finasteride OR dutasteride OR 5-ARIs).

### 2.2 Eligibility criteria

Studies that met the following PICOS criteria were included:

- Patients: diagnosed with BPH or AGA according to ICD-10 (International Classification of Diseases, 10th Revision) where information was available;
- Intervention: treatment with 5-ARIs, including finasteride and dutasteride;
- Comparison: none or non-5-ARIs (standard of care for BPH/AGA or another intervention);
- Outcome: suicide and neuropsychiatric events, including anxiety, depression and intentional self-harm (ISH);
- Study: observational studies, both prospective and retrospective, including cohort studies and case-control studies, analyzing the safety profile in a real-life context of 5-ARIs.

Clinical trials, preclinical studies, case-reports/series, reviews, meta-analyses, comment/letter/opinions, abstracts and guidelines were excluded. Articles not in English language and observational studies evaluating a different population or not considering outcomes of interest of this article were excluded as well. Study eligibility was assessed by two independent reviewers (FF and CP). Any disagreements were resolved by consulting two additional reviewers (VL and CR).

### 2.3 Data extraction

One author (FF) independently extracted data from the included studies, and a second author (CP) verified them. The extracted data was collected into a Microsoft Office Excel spreadsheet. From each retrieved article, the following data were extracted: first author, year of publication, country, study design, total number of enrolled patients and proportion of men, age of enrolled patients, number of arms, treatments and comparators for each study, therapeutic indications of 5-ARIs treatment, time of follow up or drug exposure, outcome of interest evaluated in the study and number of outcome events, and hazard ratio (HR)/Risk Ratio (RR) with its 95% confidence interval (CI) for the outcomes of interest. For each case-control study included in our meta-analysis data on case and control definition were collected.

### 2.4 Endpoint and statistical analysis

The primary outcome was suicide, the secondary outcomes were other neuropsychiatric events, including anxiety, depression and ISH. For both outcomes, the endpoints considered were the number of patients who died by suicide or experienced the neuropsychiatric events of interest and RR or HR with its 95%CI, from the beginning of the study till its end or the last follow-up available. For studies reporting data from different Follow-up (FU), the most recent was taken for forest plot analyses. A random effect meta-analysis approach was used, with heterogeneity of the effect across studies assessed by using the Cochran’s Q statistic. A P value of Q statistic < 0.10 was considered significant. For multiple-arm studies, dichotomous outcomes were expressed as RRs or HRs with 95%CIs. For single-arm studies, the proportion of patients developing specific neuropsychiatric AEs for each drug was calculated, providing insights into this pattern associated with 5-ARIs inhibitors.

To quantify the percentage of total variation across studies that is due to heterogeneity rather than chance, I2 statistics were provided. I2 values less than 25%, 25–75%, or greater than 75%, were considered for low, moderate, or high heterogeneity, respectively. We set the level of statistical significance at a p value < 0.05. Publication bias was assessed visually using a funnel plot when enough studies were included in the analysis (n ≥ 10).

For double-arm studies, dichotomous outcomes were expressed as RRs or HRs with 95% CIs. For single-arm studies, the proportion of patients developing neuropsychiatric events for each drug was calculated, providing insights into this pattern associated with 5-ARIs. All statistical analyses were performed with programs STATA v.18 and Cochrane’s Review Manager (RevMan).

### 2.5 Quality assessment

Articles included in the study were evaluated for the quality of evidence by using the Newcastle-Ottawa Scale (NOS) (12), whose results were converted into Agency for Healthcare Research and Quality (AHRQ) standards reported as good, fair, and poor. The quality was defined: (1) “good” if with NOS it achieves 3 or 4 stars for the selection domain, and 1 or 2 stars for the comparability domain, and 2 or 3 stars for the outcome domain; (2) “fair” if it achieves 2 stars for the selection domain, and 1 or 2 stars for the comparability domain, and 2 or 3 stars in the outcome domain; (3) “poor” if it achieves 0 or 1 star for the selection domain, or 0 stars for the comparability domain, or 0 or 1 stars for the outcome domain.

### 2.6 Certainty of evidence assessment (GRADE)

The certainty of evidence for each outcome was assessed using the Grading of Recommendations Assessment, Development and Evaluation (GRADE) framework. The certainty of evidence was then evaluated across five domains: risk of bias, inconsistency, indirectness, imprecision, and publication bias. For each domain, the certainty rating was downgraded by one level when serious limitations were identified and by two levels when limitations were considered very serious. The overall certainty of evidence was determined separately for each outcome and classified as high, moderate, low, or very low. High-certainty evidence indicates that further research is very unlikely to change confidence in the estimated effect. Moderate-certainty evidence indicates that further research is likely to have an important impact on confidence in the estimate and may change it. Low-certainty evidence indicates limited confidence in the effect estimate, and further research is likely to have an important impact on both the estimate and confidence in it. Very low-certainty evidence indicates very limited confidence in the effect estimate and that the true effect is likely to be substantially different from the estimated estimate (13).

## 3 Results

### 3.1 Studies characteristics

A total of 4.852 records were found in the PubMed, Embase, and Web of Science (WOS) databases, of which 66 were evaluated for full text (**Figure 1**). Eleven articles (covering a population of 4.065.899 patients) were included in the systematic review, of which 9 were meta-analyzable (14–22).

**Figure 1:**
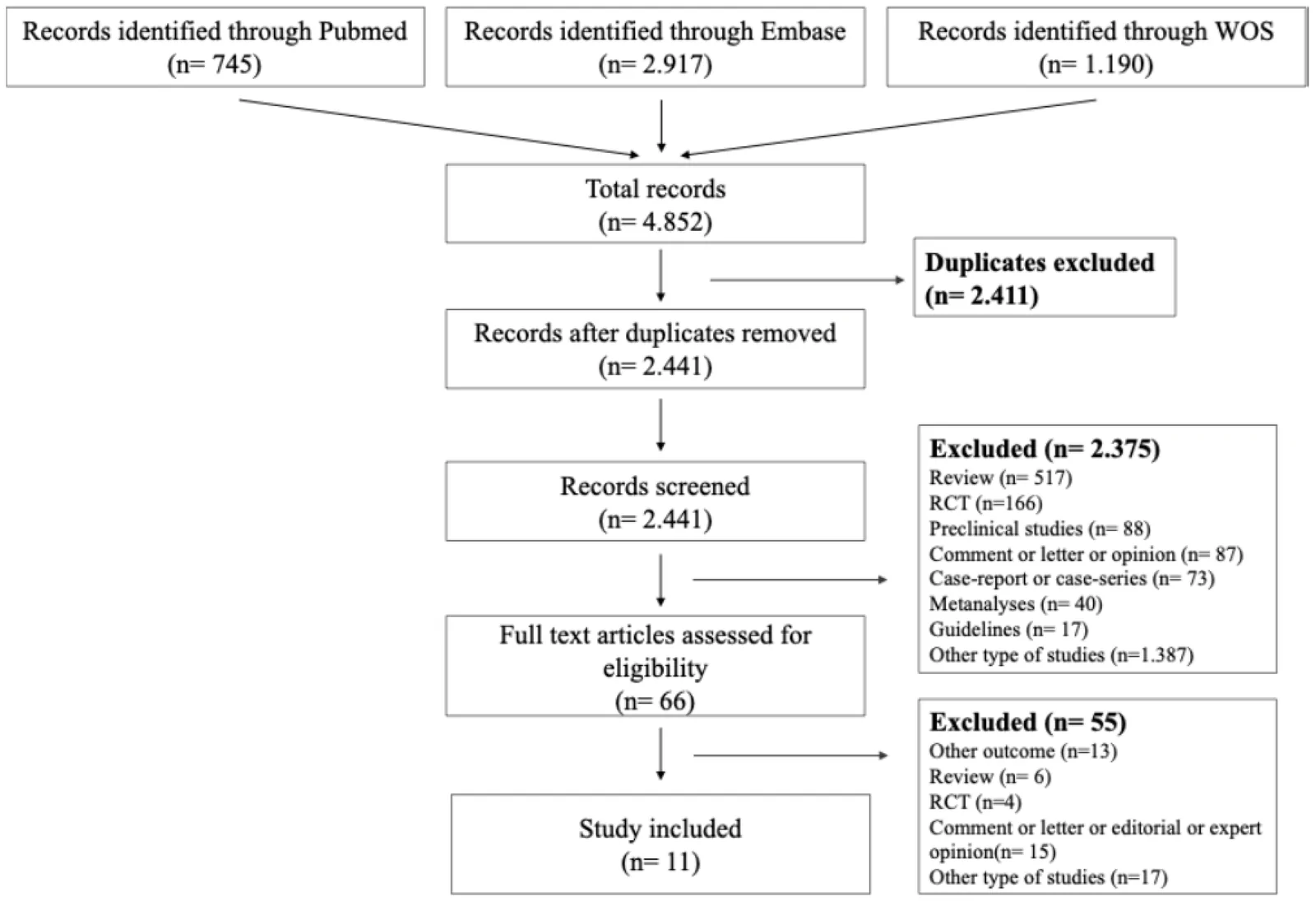
Flow diagram of the study select process.

Seven studies were cohort studies (6, 15–18, 21, 23), and 4 were case-control study (14, 19, 20, 22), mainly nested in a cohort study. Regarding the geographic distribution of included studies, studies were conducted in Asian countries, Canada and Europe, providing a global perspective on the safety profile of 5-ARIs. All studies enrolled male patients with BPH (6, 15–21), AGA (21), or both (14, 20). In most studies, patients were >45 years old. Eight studies were double-arm (14–18, 20, 22, 23), 1 was triple-arm (19), 1 was single-arm study (6) and 1 was a 5-arm study (21). An overview of the baseline characteristics of the included studies is presented in **Table 1**.

**Table 1:** Characteristics of papers included in the meta-analysis.

Study	Country	Study design	Number of patient	Age (mean ± SD)	Age (min)	Age (max)	Arms	Treatment, n (%)	Comparator	Therapeutic indication (%)	Outcome	Follow up or drug exposure (median or mean)
Jinhyun Kim, 2025 (14)	Republic of Korea	Nested case-control study	17.714	case: 69.1 ± 8.8; control: 68.5 ± 8.5	NA	NA	2	5-ARIs, 9794 (55.2)	5-ARIs never users	BPH (NA); AGA (NA)	Suicide/ISH	5.7y
Welk, 2017 (15)	Canada	Matched cohort study	186.394	75	≥ 66	NA	2	5-ARIs	5-ARIs never users	BPH (100)	Suicide/ISH/ depression	>3y
Pennap, 2024 (16)	California	Retrospective cohort study	1.032.070	75,6	≥ 65	NA	2	5-ARIs, 181.441 (17.6)	Alfa-blocker	BPH (100)	Suicide/ISH	0.85y
Jihoon, 2020 (17)	Republic of Korea	Retrospective cohort study	51.466	NA	≥ 60	NA	2	5-ARIs, 5.445 (10.6)	5-ARIs never users	BPH (100)	Suicide	7y
Yeon, 2022 (18)	Republic of Korea	Retrospective cohort study	20.111	57.5 ± 10.4	NA	NA	2	5-ARIs 1.461 (7.2)	Alfa-blocker	BPH (100)	Depression/ anxiety disorders	NA
Hagberg, 2017 (19)	Canada	Nested case-control study	14.175	case: 69.1 ± 12.8; control: 69.2 ± 12.4	18	≥ 90	3	5-ARIs	Alfa-blocker Alfa-blocker+5-ARIs	BPH (100)	Depression	NA
Lyakhovitsky, 2024 (20)	Israel	Case-control study	64.196	BPH group: 61.5 ± 17.4; AGA group: 31.4 ± 10.3	NA	NA	2	Finasteride, 23.534 (36.6)	Finasteride never users	BPH (1.3); AGA (98.7)	Depression/anxiety/ psychoses	NA
Laanani, 2023 ^NM^ (23)	France	Cohort study	287.363	NA	≥ 50	NA	2	Finasteride, 69.786 (24.2)	Dutasteride	BPH (100)	Suicide/ISH	≥ 6m
Garcia-Argibay, 2022 ^NM^ (21)	Sweden	Cohort study	2.236.876	55§	50	90	5	Finasteride 70645 (3.5), Dutasteride 8582 (0.38)	Unexposed Alfa-blocker Alfa-blocker+5-ARIs	BPH (100)	Suicide/depression	≥ 1-6m
Choi, 2016 ^NM^ (24)	Republic of Korea	Cohort study	712	29.3 ± 6.0	18	41	1	Dutasteride, 712 (100)	-	AGA (100)	Depression/ psychiatric disorders	NA
Clifford, 2002 (22)	England	Nested case-control study	154.822	NA	≥ 45	NA	2	Finasteride, 368 (0.24)	No BPH therapy	BPH (100)	Depression	6336 py

§ median age; NM, not included in the meta-analysis.

### 3.2 Suicide

Out of 5 studies evaluating suicide/suicidal ideation as the primary outcome (14–17, 21), four were considered for the forest plot analysis (**Figure 2**). Specifically, in 3 studies 5-ARIs users were compared with non-users (14–16, 18) while in 1 study (16) 5-ARIs users were compared with alpha-blocker users. Specifically, in three studies (15–17, 23), patients were treated for BPH, and in one study (14) they were treated for both BPH and AGA. Overall, our results show no statistically significant differences between groups (HR, 1.07, 95% CI: 0.76 - 1.51), with large heterogeneity between studies (I2 = 59%, p = 0.06).

**Figure 2:**
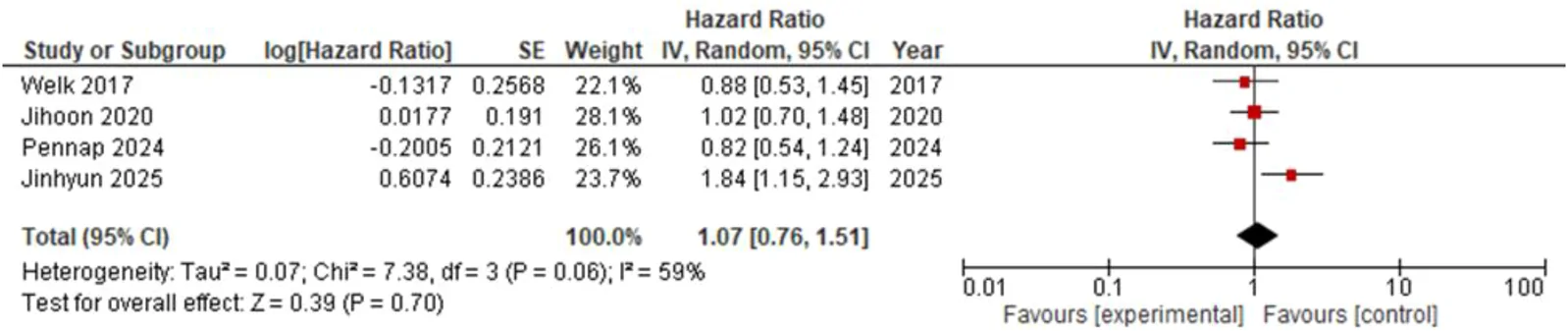
Risk of suicide or suicidal ideation in patients with benign prostatic hyperplasia (BPH) or androgenetic alopecia (AGA) for 5 – ARI compared to that of control group.

### 3.3 Neuropsychiatric events

With regard to studies that evaluated neuropsychiatric events as the primary outcome (6, 15, 18–20, 22), three studies (16–20, 23) compared 5-ARIs vs. alpha-blockers in patients treated for BPH, one study (16) compared 5-ARIs vs. 5-ARIs never users in patients with BPH, and one study (18) compared finasteride (1 mg dose, 5 mg dose) versus finasteride never users for the treatment of BPH and AGA and one finasteride vs. no BPH therapy (20, 22). Our results show no statistically significant differences between groups (HR, 1.23, 95% CI: 0.74–2.03), with very high heterogeneity between studies (I2 = 99%, p = 0.43) (**Figure 3**).

**Figure 3:**
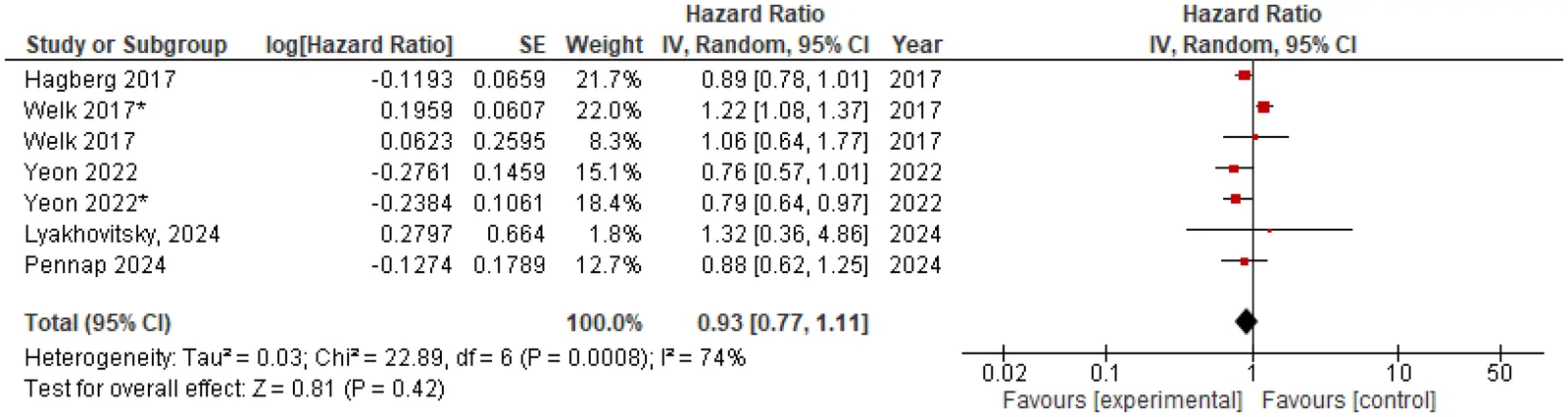
Risk of neuropsychiatric events in patients with benign prostatic hyperplasia (BPH) or androgenetic alopecia for 5 -ARI compared to that of control group neuropsychiatric events.

### 3.4 Quality of included studies

Based on the quality assessment, 6 out of 11 studies were classified as good quality, achieving 3 stars for the Selection domain, 2 stars for the Comparability domain and 3 stars for the Outcome domain/Exposure domain; 4 studies were classified as fair quality, achieving two or one stars for the Outcome domain (**Table 2**).

**Table 2:** Evaluation of the quality of included studies through the Newcastle-Ottawa Scale (NOS).

Study	Selection	Comparability	Outcome	Final results
Jinhyun Kim, 2025 (14)	***	**	***	8
Welk, 2017 (15)	***	**	***	8
Pennap, 2024 (16)	**	*	**	5
Jihoon, 2020 (17)	**	*	**	5
Yeon, 2022 (18)	***	**	***	8
Hagberg, 2017 (19)	**	*	*	4
Lyakhovitsky, 2024 (20)	***	**	***	8
Laanani, 2023 (23)	**	*	**	5
Garcia-Argibay, 2022 (21)	***	**	***	8
Choi, 2016 (24)	**	*	*	4
Clifford, 2002 (22)	***	**	***	8

According to the Newcastle-Ottawa Scale, for cohort studies, a maximum of 4 stars can be allotted for the Selection domain (that includes the item Representativeness of the Exposed Cohort, Selection of the Non-Exposed Cohort, Ascertainment of Exposure and Demonstration That Outcome of Interest Was Not Present at Start of Study), a maximum of 2 stars can be allotted in the category Comparability (Comparability of Cohorts on the Basis of the Design or Analysis) and a maximum of 3 stars can be allotted in the category Outcome (Assessment of the outcome, Was Follow-Up Long Enough for Outcomes to Occur, Adequacy of Follow Up of Cohorts). Allocation of stars defined the quality as ‘good,’ ‘fair’ or “poor. The overall score ranged from 5 to 9 points, indicating that all included studies demonstrated good or poor quality.

The symbols represent the stars awarded for each domain under the Newcastle-Ottawa Scale (NOS): Selection (*), Comparability (**) and Outcome (***).

### 3.5 Certainty of evidence (GRADE)

The results of the GRADE assessment are summarized in **Figure 4**. Overall, the certainty of evidence was very low for the majority of outcomes, with only a limited number of outcomes rated as low certainty. No outcomes reached moderate or high certainty.

**Figure 4:**
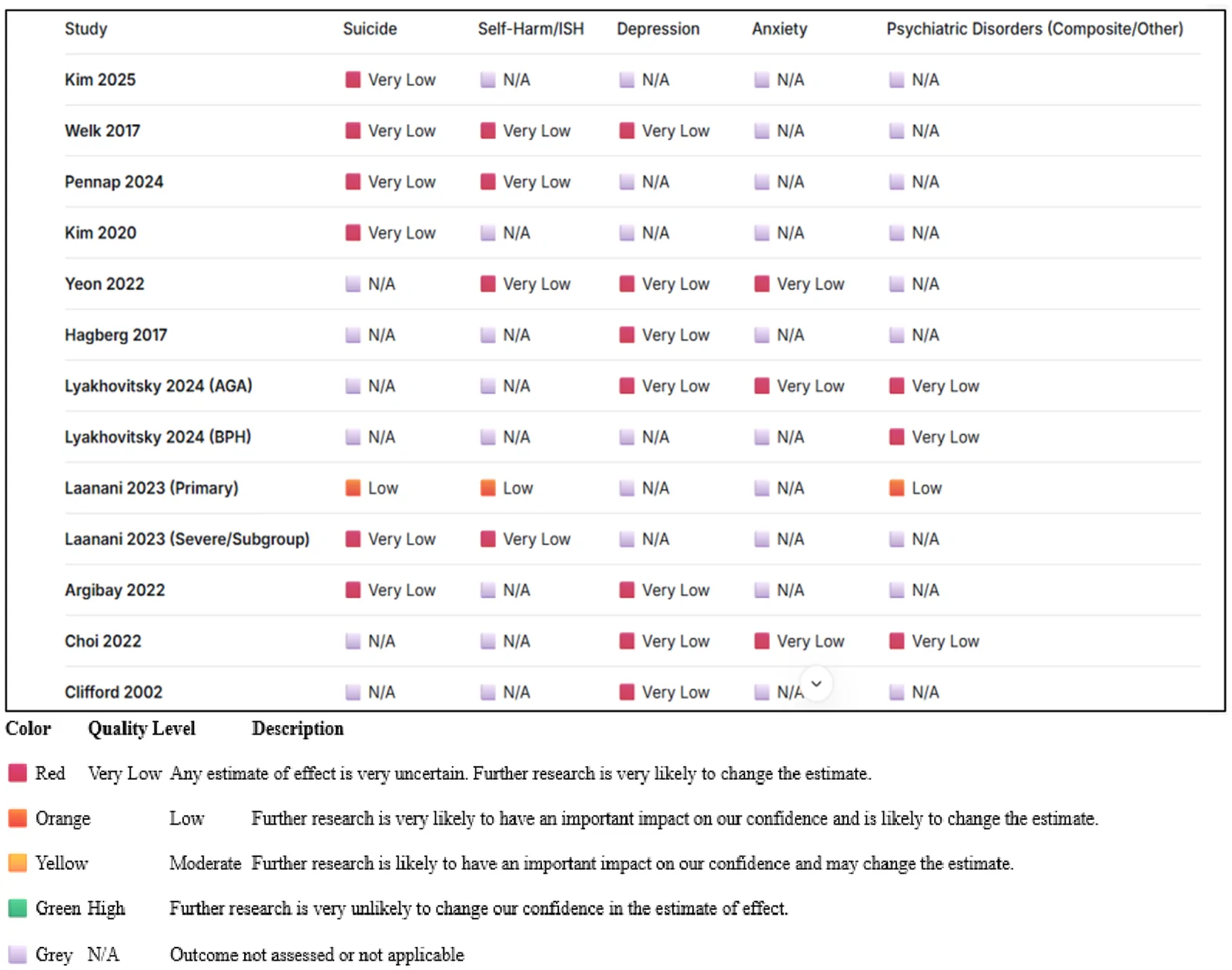
GRADE certainty of evidence across included studies and outcomes. The figure summarizes the certainty of evidence for each study-outcome pair according to the GRADE framework. Certainty was assessed at the outcome level and categorized as high, moderate, low, or very low. Downgrading was primarily driven by risk of bias, indirectness of the evidence (including reliance on observational and administrative data), and imprecision due to the rarity of events such as suicide and self-harm. Inconsistency between studies contributed to further downgrading for selected outcomes. A color-coded scheme was used to represent the different levels of certainty, with very low, low, and not available (NA) corresponding to red, orange, yellow, green high and gray, respectively, to facilitate visual interpretation.

## 4 Discussion

We carried out a systematic review and meta-analysis of 11 observational studies to define the association between 5-ARIs and the occurrence of suicide and/or neuropsychiatric AEs. Overall, our results showed no statistically significant differences between groups in most patients treated for BPH for the risk of suicide/suicidal ideation (HR, 1.07, 95% CI: 0.76–1.51). Similarly, no statistically significant differences between the groups have been found for the risk of neuropsychiatric events (HR, 1.23, 95% CI: 0.74–2.03). This is consistent with the results of a recent systematic review and meta-analysis on the association between 5-ARIs use and the risk of depression and suicide (10). In this regard, a case study focusing on young men treated for AGA describes symptoms such as severe depression, anhedonia, insomnia and, in some cases, suicidal ideation or suicide, often associated with persistent sexual dysfunction (25). This discrepancy implies that the risk may be modulated by factors such as age, indications, psychiatric history and genetic predisposition. Several reviews and observational studies have reinforced the potential association between 5-ARIs and depressive symptoms, indicating that depressive outcomes may be underreported and require careful monitoring (7). Furthermore, broader analyses of adverse effects related to 5-ARIs therapy, including sexual dysfunction, endocrine alterations, and mood changes, suggest a complex pharmacological impact that may contribute to patient vulnerability (26). The association between 5-ARIs and neuropsychiatric symptoms/suicidal behavior has already been investigated by studies reporting as a possible underlying mechanism the ability of 5-ARIs to inhibit the conversion of progesterone and testosterone into potent neurosteroids, such as allopregnanolone, a positive allosteric modulator of the GABA-A receptor. In fact, reduced levels of allopregnanolone are consistently associated with anxiety, depressive symptoms, vulnerability to stress and impaired emotional regulation. In addition, preclinical studies have shown that finasteride administration reduces neurosteroid concentrations in the brain and alters GABAergic neurotransmission, generating behavioural profiles like those described above (27, 28). Apart from neurosteroid depletion, further studies highlight the ability of 5-ARIs to penetrate the central nervous system (CNS), potentially exerting direct effects on neuronal signaling, synaptic plasticity, and hippocampal neurogenesis, mechanisms involved in mood regulation (28). Further evidence shows that post-finasteride syndrome may represent an emerging clinical condition involving persistent sexual, neuropsychiatric, and physical symptoms, even after discontinuation of the drug, as described in several clinical studies (29). Overall, the literature supports a multifactorial model in which neurosteroid dysregulation, possible epigenetic susceptibility, CNS penetration, and psychosocial factors interact to produce neuropsychiatric symptoms. Although causality is not definitively established, the mechanistic rationale is biologically plausible, and the accumulating evidence calls for cautious prescribing, particularly in younger individuals and those with psychiatric vulnerability (7, 27, 28).

Although these mechanistic hypotheses provide biological plausibility, it is important to acknowledge that much of the supporting evidence derives from preclinical studies, case reports, and small observational investigations. Therefore, these mechanisms should not be interpreted as evidence of causality and must be considered in the context of the overall epidemiological evidence. Notably, the pooled results of the present meta-analysis did not demonstrate a statistically significant increase in the risk of suicide/suicidal ideation or neuropsychiatric adverse events among men treated with 5-ARIs (30). In addition, data from studies included in our meta-analysis highlighted the presence of many alternative causes, not directly related to the pharmacological effect of finasteride or dutasteride. For example, Welk et al. demonstrated that current depressive status, past self-harm, or ongoing antidepressant treatment strongly predicted subsequent suicidal outcomes and that these underlying vulnerabilities served as powerful confounding factors even after statistical adjustments (17). This can also be seen in the study conducted by Kim et al., in which psychiatric history significantly influenced risk estimates and may partly explain the early increases in suicidality after starting 5-ARIs therapy. Another consistently highlighted factor is indication confounding, particularly when comparing patients treated for BPH with those treated for AGA (17) for which the psychosocial context substantially varies.I Indeed, younger patients with AGA may experience body image issues, reduced self-esteem, and social anxiety, all independently contributing to depressive symptoms. Lyakhovitsky et al. reported that psychiatric outcomes in finasteride users varied according to the underlying indication, with AGA patients exhibiting a different psychological profile than older patients with BPH. Choi et al. noted that the efficacy-safety ratio between dutasteride and finasteride in AGA must be interpreted within this unique psychosocial framework, which may predispose to mood alterations regardless of drug exposure (6, 20). Several large cohort studies also highlighted the impact of age, multimorbidity, and cognitive decline on the development of depressive symptoms and suicidality. Garcia-Argibay et al. observed that associations between 5-ARIs and dementia, depression, or suicide tended to diminish in the presence of comorbidities, suggesting that underlying frailty and cognitive impairment, often seen in elderly patients with BPH, may contribute more to psychiatric outcomes than the drug itself (15, 21). This interpretation is reinforced by the findings of Pennap et al., in which advanced age and medical complexity emerged as strong predictors of intentional self-harm, regardless of treatment class (16).

The temporal dynamics also provide important food for thought. Some studies describe an early clustering of psychiatric events shortly after the start of treatment, increasing the possibility of bias due to pre-existing conditions, in which prodromal symptoms of depression or suicidality precede and indirectly trigger the prescription of drugs. This phenomenon is particularly evident in the work of Hagberg et al., in which the risk of depression treated with antidepressants appeared high, mainly in the initial phase after the start of 5-ARIs therapy and subsequently decreased. Such patterns suggest that increasing psychological distress may have prompted men to seek medical assistance for urological symptoms, making the temporal association with the start of treatment misleading. Overall, these findings indicate that the relationship between 5-ARIs and suicidal tendencies is complex and influenced by multiple factors, only partially observed in the various datasets (19). Considering initial psychiatric history, indication-specific vulnerability, early treatment biases, sexual dysfunction as a mediating mechanism, and socioeconomic or health factors, many of the apparent associations described in crude or minimally adjusted analyses tend to diminish or disappear. This supports a cautious interpretation of risk signals and emphasizes the importance of comprehensive adjustment strategies in assessing psychiatric safety in men treated with 5-ARIs. Recent evidence further helps to contextualize the therapeutic positioning of 5-ARIs within the broader management of benign prostatic obstruction. Studies investigating surgical and functional outcomes in patients undergoing treatment for BPH have highlighted that lower urinary tract symptoms are frequently driven not only by prostatic enlargement but also by concomitant bladder dysfunction, including detrusor underactivity and impaired bladder compliance. In this context, evidence from clinical studies (31, 32) shows that postoperative functional outcomes are strongly influenced by preoperative bladder function and the degree of lower urinary tract remodeling. These findings emphasize that the management of BPH should be interpreted within a bladder–prostate functional unit rather than a prostate-centric model alone. Accordingly, when medical therapy with 5-ARIs is limited by adverse effects, inadequate response, or patient preference, escalation to surgical or minimally invasive interventions should be guided by a comprehensive functional assessment, including urodynamic parameters where appropriate. This integrated approach allows more accurate patient selection and improves prediction of postoperative outcomes, particularly in individuals with suspected bladder dysfunction.

These observations suggest that a substantial proportion of the reported associations may be explained by residual confounding, indication-related factors, pre-existing psychiatric vulnerability, and differences in patient characteristics rather than a direct pharmacological effect of 5-ARIs. This interpretation is consistent with the overall findings of our meta-analysis, which did not identify a significant increase in psychiatric risk associated with 5-ARI exposure (33).

Several recently published case reports and post-marketing case series have examined the potential association between 5-ARIs and the onset of adverse psychiatric events, including suicidal behavior. Li et al. reported on three young male patients who developed persistent post-finasteride syndrome (PFS) following its use for androgenetic alopecia (34). In addition to sexual dysfunction, the patients exhibited neuropsychiatric symptoms such as anxiety, depressive traits, insomnia, and social withdrawal. The same study suggests that genetic predisposition may play a role in the persistence and severity of psychiatric manifestations after discontinuation of the drug (PFS) (35). Similarly, Gupta et al. described what the authors termed an atypical and irreversible form of post-finasteride syndrome. In this case, neuropsychiatric symptoms coexisted with systemic and sexual dysfunction in a complex clinical picture that was difficult to explain (3). The authors highlighted the pharmacological complexity of adverse events induced by 5-ARIs and the absence of a robust mechanistic model to explain why only a minority of exposed individuals developed long-lasting neuropsychiatric complications. The series published by Irwig et al. adds an even more worrying picture. By examining post-marketing reports of men who developed severe psychiatric symptoms leading to suicidal ideation and, in some cases, complete suicide, the study raises the hypothesis that, for a subset of susceptible patients, persistent sexual dysfunction combined with sleep disturbances and affective symptoms may precede suicidal behavior (35). Although the report does not provide evidence of causality, it reinforces the concept of a possible clinically relevant association, particularly in individuals who develop long-lasting adverse events after discontinuing treatment. Together, these three publications illustrate the growing concern about the psychiatric safety profile of finasteride, in addition to its known sexual adverse effects. However, these findings primarily originate from case reports and post-marketing observations, which are inherently limited in their ability to establish causality. Consequently, while such reports warrant clinical awareness and further investigation, they should be interpreted cautiously and weighed against the overall findings from larger observational studies and pooled analyses. Despite the absence of a significant association observed in our pooled analyses, regulatory agencies continue to monitor the psychiatric safety profile of 5-ARIs. Following an EU-wide review of available data, the EMA has advised that patients receiving finasteride should discontinue treatment and seek medical advice if they experience depressed mood, depression, or suicidal ideation. Similarly, contemporary guidelines for the management of male lower urinary tract symptoms acknowledge these potential psychiatric adverse effects and recommend appropriate patient counselling and monitoring, particularly in individuals with a history of psychiatric disorders.

Our meta-analysis has some limitations, such as the presence of studies with different characteristics, particularly in terms of sample size, geographical distribution and follow-up duration, which may have influenced our results. Overall, heterogeneity was high (>60%) for all analyses. Included studies differed with respect to indication (BPH versus AGA), age distribution, comparator groups, exposure duration, outcome definitions, and type of 5-ARI used. Furthermore, the composite category of neuropsychiatric adverse events encompassed clinically distinct outcomes, including depression, anxiety, psychosis, intentional self-harm, and suicidal behavior. Since all included studies were observational, the results remain susceptible to residual confounding, indication bias, and outcome misclassification. Finally, publication bias cannot be completely excluded.

This could be related to multiple factors, including the limited sample size and power of the included studies, which may have led to less precise estimates, and their results may be more susceptible to random variation, significantly increasing heterogeneity. Despite these limitations, using three databases (PubMed, Embase, and Web of Science), we conducted a systematic review and meta-analysis of observational studies, providing an updated overview of the safety profile of 5-ARIs in terms of suicide as the primary outcome and other neuropsychiatric events, including anxiety, depression and intentional self-harm as secondary outcomes. For both primary and secondary outcomes, the endpoints considered were the number of patients who died by suicide or who experienced the neuropsychiatric events of interest.

## 5 Conclusions

This meta-analysis provides valuable information on the risk of adverse neuropsychiatric events associated with 5-ARIs in patients with BPH or AGA compared with none or non-5-ARIs (standard of care or another intervention). No difference was found in the risk of developing events such as suicide and neuropsychiatric events, including anxiety, depression and intentional self-harm (ISH). However, the PRAC verified that suicidal thoughts are a negative effect of 5-ARIs and suggested measures to lower the risk of suicidal ideation associated with finasteride and dutasteride by reviewing the available data on finasteride tablets across the EU. Based on this consideration, we believe it is advisable to continue monitoring patients taking these drugs in real-life settings through active surveillance programs and *ad hoc* prospective observational studies (36, 37) to specifically monitor the onset of suicide and neuropsychiatric events. Additionally, the broader therapeutic positioning and safety profile of 5-ARIs, including their long-term effects on prostate cancer risk in patients with benign prostatic hyperplasia, have been extensively evaluated in recent large-scale database analyses and clinical trials (38, 39).

## Data Availability

The original contributions presented in the study are included in the article/supplementary material. Further inquiries can be directed to the corresponding author.
